# Development of the infant foot as a load bearing structure: study protocol for a longitudinal evaluation (the Small Steps study)

**DOI:** 10.1186/s13047-018-0273-2

**Published:** 2018-06-20

**Authors:** Carina Price, Juliet McClymont, Farina Hashmi, Stewart C. Morrison, Christopher Nester

**Affiliations:** 1 Centre for Health Sciences Research, University of Salford, Frederick Road, Salford, UK; 2 School of Health Sciences, University of Brighton, Darley Road, Eastbourne, UK

**Keywords:** Infant biomechanics, Foot development, Milestone, Infant skin

## Abstract

**Background:**

An improved understanding of the structural and functional development of the paediatric foot is fundamental to a strong theoretical framework for health professionals and scientists. An infant’s transition from sitting, through crawling and cruising, to walking is when the structures and function of the foot must adapt to bearing load. The adaptation of skin and other hard and soft tissue, and foot and gait biomechanics, during this time is poorly understood. This is because data characterising the foot tissue and loading pre-walking onset does not exist. Of the existing kinematic and plantar pressure data, few studies have collected data which reflects the real-life activities of infants with modern equipment.

**Methods:**

This is a longitudinal study and part of the Great Foundations Initiative, a collaborative project between the University of Brighton and the University of Salford, which is seeking to improve foot health in children. Two cohorts of 50 infants will be recruited at the two sites (University of Brighton, Eastbourne, UK and University of Salford, Salford, UK). Infants will be recruited when they first reach for their feet and attend four laboratory visits at milestones related to foot loading, with experienced independent walking being the final milestone. Data collection will include tissue characteristics (skin thickness, texture, elasticity, pH and tendon thickness and cross-sectional area), plantar pressures and kinematics captured during real world locomotion tasks.

**Discussion:**

This study will provide a database characterising the development of the infant foot as it becomes a weight bearing structure. The data will allow effective comparison and quantification of changes in structure and function due to maturation and loading by measuring pre and post established walking. Additional variables which impact on the development of the foot (gender, ethnicity and body weight) will also be factored into our analysis. This will help us to advance understanding of the determinants of foot development in early childhood.

## Background

The stages of infant development from sitting to crawling and eventually independent walking represent a series of important motor control and movement co-ordination milestones [1–3]. This is the period during which the foot becomes essential for balance, stability and locomotion and therefore central to enabling a child to learn about and engage with their social and physical environments. Foot structures bear load for the first time and progressively adapt to support the entire body weight regularly, cyclically and across different terrains and obstacles. Changes to the dimensions, shape and structure of the feet occur throughout infancy and childhood [4, 5]. However, detailed changes to foot morphology (e.g. more resolution than simply width and length), structure (e.g. tissue characteristics) and function (e.g. gait and pressure), are yet to be quantified during this stage of life, however, especially through longitudinal studies.

Precise knowledge of the development of the paediatric foot and gait is essential to underpin paradigms for clinical practice and locomotion research. However, having very young participants in a gait laboratory is not a trivial task. There are specific ethical and governance requirements (e.g. safe guarding and consent), as well as challenges in terms of the size of the infant, issues of walking trajectory, consistency of movement tasks and the ability of the infant to be directed and follow instructions. Existing literature on gait development has employed both cross-sectional [6, 7] and longitudinal study designs [8–11]. However, studies generally begin once weight-bearing has commenced, therefore missing the alterations to foot structure and function that occur with the initiation of weight-bearing. Data exists from early walkers (0–5 months of walking experience) including simple temporal-spatial characteristics and some quantification of limb and foot function in the form of plantar pressure, kinematics, electromyography and kinetics [12–14].

Undertaking standardised gait protocols with infants has proved demanding, with Bertsch et al., 2004 collecting 40–50 walking trials per participant to obtain five suitable plantar pressure data sets [14]. Furthermore, all existing data has been collected within an experimental gait laboratory environment and using protocols which involve leading the infant by the hand, or encouraging them to walk in straight lines [14]. The external validity of these approaches has not been reviewed, but they contrast with an infant’s real-world movements. Specifically, the field of developmental psychology shows us that novice walker infants rarely walk in straight lines, preferring a few steps at a time and in multiple directions [15, 16]. It also demonstrates that this variability is a key feature of motor learning and should therefore be included in our experimental design. This contrasts with existing approaches and indeed this may explain why Bertsch and colleagues had to collect so many trials to achieve straight line walking.

Whilst some aspects of gait have been studied in infants, changes in foot soft tissues due to the commencement of weight bearing have not been documented in longitudinal studies. It is not known whether this phase of infant development would significantly influence the properties of the plantar tissues and the toe nails. Current literature infers changes to skin hydration, viscoelastic properties and surface texture with age [17–19], in addition to age related differences in skin thickness and composition [18, 20]. Differences in skin characteristics between different sites on the foot are evident in adults, for example dorsal v plantar skin; plantar surface of the metatarsal head versus medial arch skin [21]. The neuromuscular development of the infant is also key; bone growth stimulates muscular adaptation as does increased loading [22]. This is supported by a physiological feedback loop (the Mechanostat) whereby osteogenesis is stimulated by strain between soft tissue and bone as a consequence of movement [23]. The properties of the Achilles tendon and plantar fascia has additionally been explored due to the suggestion that the two are continuous in infants [24]. There also exists a hypothesis that the curvature of nails is due to mechanical forces, but this has not been explored in infants or in the feet as opposed to the hands [25, 26].

In response to this lack of knowledge and information relating to the paediatric foot, the aim of our research is to investigate the changes that occur in the infant foot as it develops from a non-weight bearing to a weight bearing structure. This is part of a wider research initiative (Great Foundations) which is also seeking to understand how parents, professional and industry view children’s foot health.

## Methods/design

### Study design

This is a two site, longitudinal study to quantify the changes in foot skin tissues, plantar pressures and lower limb motion in infants. The study will recruit participants prior to walking onset and involves them attending for four visits, defined to reflect the following stages of foot loading:

- Visit 1. Baby can grab their feet for the first time when lying on their back
- Visit 2. Baby can pull to stand for first time using furniture and with no external assistance
- Visit 3. Baby can stand unsupported and take up to five independent steps
- Visit 4. Baby is a confident, balanced stable walker and can pick things up, hold things and communicate while walking

The intention of this work is not to define gross motor development via milestone achievement but define foot-specific loading tasks in infancy. This approach hasn’t previously been defined in the literature but, through undertaking this approach, we are looking to re-frame how we define foot development and advance our understanding of the development of the foot as a weight-bearing structure.

Ethical approval has been obtained from the ethics committee of the School of Health Sciences University of Salford (approval number HSCR161779) and the School of Health Sciences University of Brighton (LHPSCREC 17–11).

### Participants

The aim of our recruitment is to span a representative population of the UK through recruitment across a range of groups of infants in the North and South of the country. Recruitment of infants will take place from a wide range of sources using a range of media. This includes poster and flyer distribution to local nurseries, play centres and baby support groups, as well as using social media to (e.g. Facebook, Twitter) to display and advertise recruitment posters. Researchers will also attend local schools, baby groups, centres, and nurseries to talk about the project to attract interest. Local radio and newspaper adverts at each site will also be employed and word-of-mouth is also expected to attract participants through snowball sampling.

Parents interested in the study will contact the primary researcher (CP or JMc) and will be screened for eligibility. Respondents who are suitable for the study will be invited to visit the baby lab. To be included in the study infants must:

1. Be born within weeks 37–42 of pregnancy and meet the development criteria for each visit.
2. Have a self-reported typical post birth screening for neurological and musculoskeletal problems.
3. Have no indicators for visual, audio, sensory or other developmental impairment at point of recruitment.
4. Be above the 4th percentile for weight at birth and at recruitment
5. Reach visit one milestone (*Flexing body lying on back bare feet: Gently bend both legs toward head 3 times. Encourage the child to grasp their feet “get your feet”*) at < 10 months of age.

Exclusion criteria: participants must not,

1. Have a familial history (siblings, parents) of inherited neurological or musculoskeletal conditions (e.g. juvenile chronic arthritis, rheumatoid arthritis, Charcot Marie Tooth).
2. Have current general skin disorders affecting the foot such as dermatitis, psoriasis or any skin abrasions.
3. Have been referred for medical/health care assessment for any suspected neurological or musculoskeletal conditions (an indicator or other significant health issue).
4. Be receiving prescription medicine (an indicator of other significant health issue).
5. Attain Visit 3 (stepping), and Visit 4 (walking 10 ft. with narrow base of support and reciprocal pattern) later than 6 months after the expected attainment date (12 and 16 months respectively).

Criteria will be parent-reported and checked by the research teams during laboratory visits and parents will be encouraged to check their infant’s heath record for information relating to inclusion criteria. Participants must pass this screening at each visit to be included in the final 4 visit study.

Following email or telephone screening the parent/guardian of the participant will be provided with an information pack to highlight the visit milestones and will await the infant achieving this before attending for their first visit. Upon achievement of each milestone, parent/guardians will contact the researcher (ideally providing a dated video or photo) and a visit will be booked as early as possible, within a maximum of two weeks. Parents/guardians will be provided with further information specific to the visit. Identification of milestones for visits 2–4 follows the same process.

### Testing procedure

Infants will attend the laboratory with a parent/guardian and be accompanied throughout the session. Laboratory areas are private with seating areas, toys and nappy changing and feeding facilities with an area for skin testing and an area for pressure and kinematics testing. The parent/guardian will be advised of the procedures during the data collection process and will be entitled to stop the researcher at any point. Time for breaks, feeding and play will be included at any stage. The parent/guardian will provide consent on behalf of the infant on arrival and each participant will be assigned a consecutive number and their left or right foot will be selected at random utilising a random number generator (which will be tested throughout all 4 visits). Any participant who is too upset or stressed by the testing will be deemed to have removed consent and testing will cease.

There will be a 20-min acclimatisation period at the start of each data collection visit, during which footwear and hosiery will be removed and the participant will play or rest in the laboratory area. The temperature and humidity of the room will be recorded and managed within set boundaries (18–25 °C and 40–60% respectively).

A questionnaire will detail participant characteristics such as days from milestone achievement and potentially related or confounding factors such as baby walker use, footwear use and time since last bath.

### Foot morphology

Sitting comfortably, and barefoot, the participant’s foot length and circumference will be quantified using a Brannock device designed for use with infants. The forefoot width will also be measured between the most medial and lateral aspects of the plantar foot in the forefoot with a flexible tape.

### Skin, tissue and nail characteristics

**Fig. 1:**
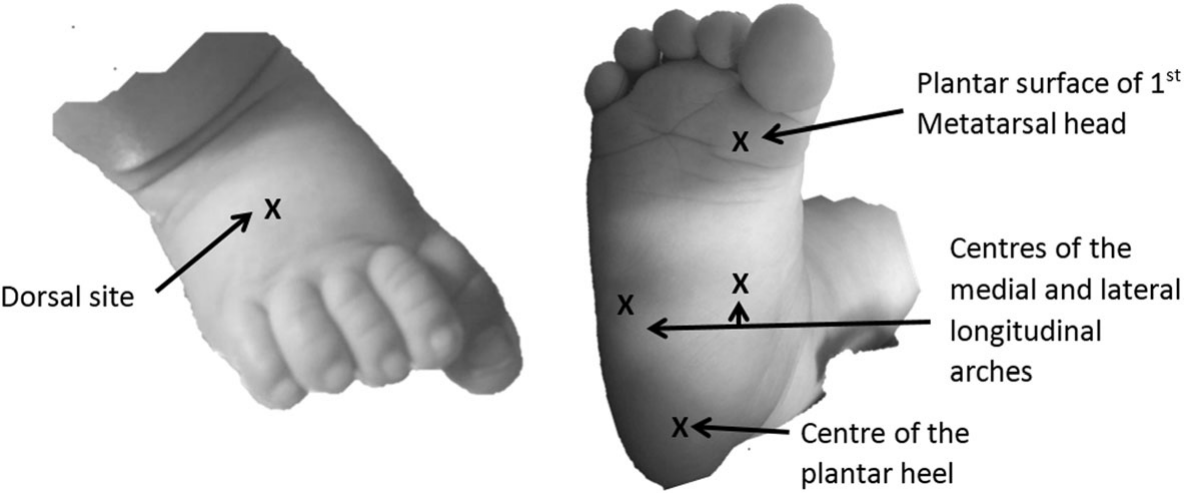
Sites on the dorsal (Left) and plantar (Right) aspect of the foot for measurement

The participant will be seated with legs extended and plantar aspects of both feet facing the investigator, in an infant feeding chair, or in the arms of the parent/guardian. Five sites will be measured [21, 27] (Fig. 1). The skin, tissue and nail measures are defined in Table 1.

**Table 1:** Skin testing measures, devices and protocols

Characteristic	Device	Methodology	Data outcome
Skin surface texture	Visioscan® VC 98 (Courage-Khazaka, Cologne, Germany)	The camera aperture will be placed over the skin site aligned to the longitudinal axis of the foot and	3 images per site.
Skin surface hydration	Corneometer® CM 825 probe (0.9–1.2 MHz, Accuracy: ±3%) (Courage-Khazaka, Cologne, Germany)	Applied perpendicular to the skin with a constant low pressure for 3 measures per site with 5 s duration left between measures	3 measures per site.
Skin elasticity	Dermascan Elasticity Probe (10 mm suction diameter) (Cortex, Hadsund, Denmark).	The suction probe will be affixed to the skin (with hypoallergenic tape supplied with the device) at the measurement sites and the cable supported by the researcher to reduce interference from the participant.	3 measures per site.
Skin pH	Dermascan PH Probe (range 1–11 pH, accuracy 0.1 pH at 25 °C) (Cortex, Hadsund, Denmark).	The probe is placed gently on the foot for approximately 10 s while a measure is recorded.	3 measures per site rear-, fore-and dorsal foot only
Skin thickness	Dermascan B-mode Ultrasound Probe (5–35 MHz, max penetration depth 3.37 mm) (Cortex, Hadsund, Denmark).	Deionised water will be applied to the probe to facilitate the positioning/movement of the probe and transmission of the ultrasound waves. Measures are taken with the probe perpendicular to the skin surface and gain range 6–10.	3 measures per site.
Tendon thickness and cross-sectional area	Venue 40 Ultrasound machine; hockey stick probe (7.6–10.7 MHz, penetration depth 3 cm) (GE Healthcare, Amersham, United Kingdom).	The participant will be held prone (with parent standing) such that the plantar surface of the foot and the Achilles tendon are available. Water based gel will be utilised and the thickness and the cross-sectional area of the Achilles quantified at a set distance from the top of the calcaneus with longitudinal and transverse measures.	3 images per orientation.
Nail shape	Camera	The participant will be sat such that the experimenter can take a photograph of the distal end of the toe unloaded.	3 images of hallux nail.

### Static tasks

A nursery- style environment of approximately 4 m^2^ has been developed at each site, with foam flooring surrounded by baby gating. A pressure platform (100 Hz; Novel Emed-xl, Germany), electromyography (EMG)/accelerometer system (2000 Hz; Delsys Trigno, Delsys inc., Boston, USA), HD video (University of Brighton: Vicon Bonita 720c video camera; University of Salford: HD Pro Webcam, Logitech) and motion capture cameras (University of Brighton 100 Hz, 6 x Vicon Vantage 5, Vicon, Oxford, UK; University of Salford: 100 Hz; 6 x Qualisys Oqus, Qualisys, Gothenburg, Sweden) will be synchronised to capture locomotion data.

To enable us to obtain plantar pressure patterns for participants across all developmental stages we will be collecting “static” pressure data during tasks relevant to each developmental stage. For this the infant will undertake tasks on the pressure platform as described in Table 2. The infant will also have a Delsys Trigno sensor (accelerometer) attached to their trunk for visits 2–4 at 60% of body length from the head to the heel.

**Table 2:** Static plantar pressure tasks for each visit

Task	Time	Visits
Standing with parent supporting infant weight under the arms round the thorax	3 × up to 30 s trials	1
Standing weight-bearing holding parent (or researcher) hands to maintain stability.	3 × up to 30 s trials	1,2
Pull to stand task	3 × up to 30 s trials	2
Standing using furniture support	3 × up to 30 s trials	2,3
Standing independently	3 × up to 30 s trials	3,4

These trials will be recorded via the plantar pressure software alongside two synchronised video cameras to enable coding the steps in terms of the movement occurring.

### Dynamic tasks

For visits 3 and 4, and reflecting the emerging walking skills, a dynamic data collection will also be undertaken. These will quantify the nature of the foot loading in terms of plantar pressure and lower limb motion.

**Fig. 2:**
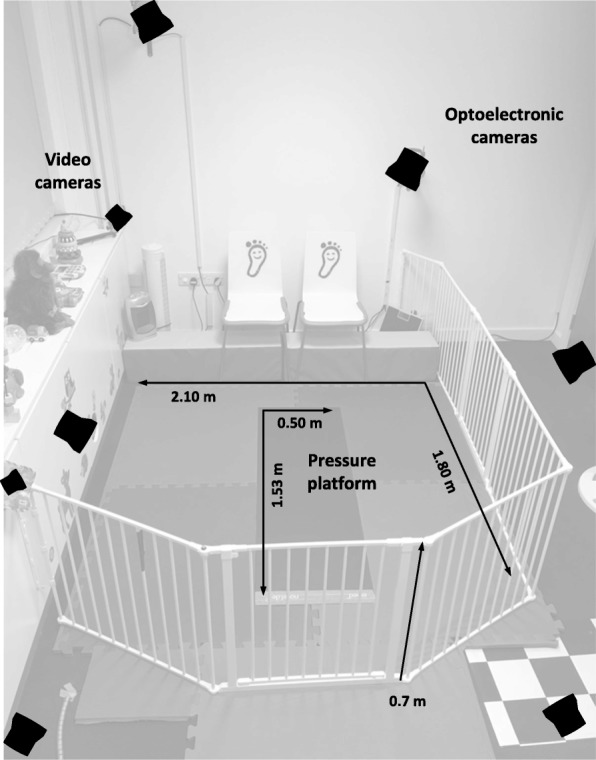
Schematic of laboratory setup including pressure platform, motion capture cameras and volume of nursery area

**Fig. 3:**
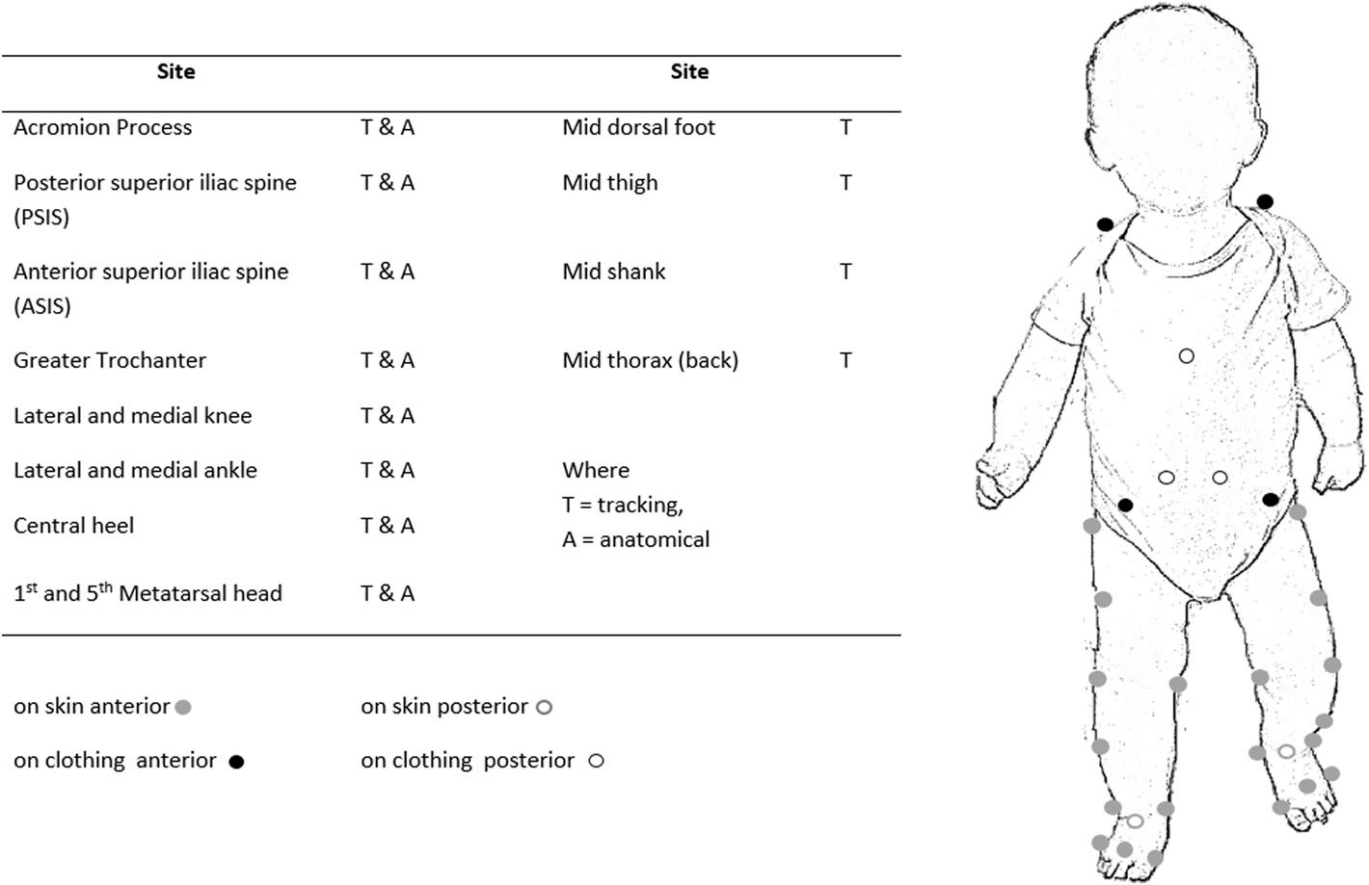
Marker placement for kinematic data collection

To collect motion data retro-reflective markers (20 X 6 mm) will be attached to anatomical sites (Fig. 2). Markers on will be positioned with hypoallergenic adhesives suitable for infant skin apart from those on the pelvis and thorax, which will be adhered to the clothing (Fig. 3).

The participant will also be instrumented with two Delsys Trigno sensors, positioned on the anterior and posterior aspects of the lower limb (at 40% of the distance from the medial knee and malleoli). The skin will be prepared by cleansing with an alcohol wipe. The EMG/accelerometer sensors will be affixed with an adhesive sticker cut from neo-natal hypoallergenic tape to match the dimensions of that supplied by the EMG system manufacturer. Following application these sensors will be wrapped with a cotton sleeve on the lower limb to prevent the infant touching the sensors and to provide further adherence.

For the dynamic task, the participant will be positioned in the data capture volume for up to 10 min with pressure, motion, EMG/accelerometer and video data collected synchronously for the entire duration. Within this period the protocol allows for free play and movement. A second period will involve encouraging the participant to move. Investigators or parents will encourage the participant to walk from one end of the testing space to the other over the pressure platform. If the participant (particularly visit 3) requires support during this time then this will be provided by gentle support from the fingers of the parent above the participant.

### Data collection and management

For the skin and tissue data, measures will be exported from the DermaLab Combo device and will include skin pH, skin thickness, Young’s Modulus, skin retraction time and viscoelasticity. The average of three measures per foot site will be computed. The Visioscan system provides arbitrary measures of smoothness, roughness and scaliness, which will be complemented with analysis in Matlab (MathWorks, Natick, USA). Photographs of the nail will be digitised and the integral and dimensions computed as defined above using a bespoke code in Matlab. Discrete variables will be computed from skin and nail data will be computed to define a mean and standard deviation (or non-parametric alternatives) for each infant for each skin site tested. Musculoskeletal ultrasound data will be measured for thickness and cross-sectional area of the Achilles tendon, taken 1 cm (cranially) from the calcaneus.

For the static and dynamic tasks data, pressure data will be manually extracted as pressure records step by step (utilising Novel Emed software, GmbH, Munich, Germany) and data exported as an ascii file with a frame for each sample. These will be analysed using Matlab and results compared and interpreted from pedobaragraphic statistical parametric maps (pSPM) [28]. Video data will be utilised to categorise the specific nature of the stepping tasks performed as, unlike existing literature, all full-contact steps will be analysed, not just those which constitute walking in a straight line.

Kinematic data will be labelled and exported for analysis in Visual 3D (C-Motion, Inc., Rockville, MD, USA). A model will enable the quantification of lower limb joint angles and body position, and locomotor speed relative to the laboratory area. The definition of the shank and foot are essential and key to the research questions and the definition of the thigh, trunk and pelvis offer the ability to categorise the motion of the infant further e.g. to quantify a locomotor velocity, or to define sitting and standing tasks. The categorisation of movement tasks is also key to interpretation of the outcome measures within this research project akin to the research questions. Accelerometer and EMG data will be incorporated within Visual 3D also and processed to consider stability co-contraction and onset timings respectively.

### Statistical analysis

Study planning has focussed on achieving 100 participants with 50 participants at each site. A power analysis will be undertaken on the pilot data, which included a 5 person repeatability study at each site for visit 4 infants.

The overriding statistical approach is to compare data across the four different milestones relating to foot loading. Primary approaches will compare mean (or median if non-parametric) data for discrete variables such as skin and tissue parameters across the four longitudinal visits using repeated measures ANOVA. More complex models will undertake correlations and also include extraneous factors which may have influenced the dependent variables such as; two-way ANOVA to compare how changes in the selected foot structure and foot function parameters across the milestones differ between girls and boys and ANCOVA to test to investigate the influence of weight, ethnicity, and age on dependent variables at attainment of each milestone. Consideration of the management of non-independent data sets, such as left and right feet from the same participant, will be imperative and the statistical approach for this will be determined on a research question basis.

## Discussion

The study described in this paper will aim to quantify the development of skin, pressure and motion parameters of the paediatric foot during real world tasks.

The external validity and clinical relevance of our research are both highly influenced by the number and timing of the longitudinal visits and therefore how they are defined. The number of longitudinal visits was chosen based on feasibility in terms of asking parents and infants to commit to a long-term study with regular visits. Four was deemed an acceptable number of visits to ask volunteers to attend laboratory spaces for two-hour test sessions over approximately a nine month period. The timing of the visits could be defined by milestone, age or set duration from the first visit. Existing research considering infant gait biomechanics either categorises infants by their age [29] or by attainment of a specific milestone [9, 10]. After significant research and debate we have selected to define infants by milestone. Existing literature shows us that age is not a suitable criteria for differentiation of biomechanical variables of infants, due to high variation in attainment of milestones [30, 31]. We have selected milestones that reflect foot loading specifically, rather than any pre-existing developmental scale. These relate directly to our research questions and are chosen with the assumption that these will relate to the amount of loading that has been undertaken before and between visits. They do not however, represent explicitly the amount of activity between visits as an infant who walks one day may not walk again the next. Age is also a confounding aspect of participants as we can expect changes to the skin characteristics with aging alone in this cohort [17, 19, 32]. To aid the interpretation of our data we will record age and corrected age (both in days) at each visit in addition to duration from milestones (in days).

The intention of the study to incorporate and define a cohort of infants which represent a typical development of babies within the United Kingdom (UK). The two-site design (in the North and South of the UK) enables the capacity to recruit participants which span the demographic make-up of the UK population. Those inclusion criteria relating to milestone attainment aim to reduce the risk of including children whose development later falls outside the typical boundaries.

Inclusion and visit timing relies on parent-reported information. To ensure this information is sound parents will be encouraged to check the child’s ‘Personal Child Health Record’ (supplied by the National Health Service at birth in the UK) and also to send video records to the researcher regularly to identify milestone achievement. Guides and regular phone calls are scheduled to inform parents as much as possible, which have been developed through pilot work and checked for comprehension by groups of parents. The questionnaire at the start of the laboratory visit also provides a further opportunity to discuss and define dates at which milestones were attained and to gather information relating to foot loading prior to visit one and between later longitudinal visits.

The collection of data from infants is challenging for many reasons including the researcher being unable to instruct them to do a task and their inconsistent locomotion choices. The infant walkers at visit three are likely to not want to walk during the test sessions and to opt to crawl when encouraged to move around the laboratory area. Our methods are flexible to allow supported walking for this visit if the infant does not voluntarily walk alone. This is a modified approach in attempt to incorporate motion strategies within the data collection which have high external validity and keeping in mind that bouts of infant walking are most commonly short in terms of steps [15]. This enables us to fully capture the changes in locomotor strategy and the trajectory of the inter- and intra-individual variability across visits [2]. It does mean that we need to consider the comparison of our data across milestones carefully as existing literature demonstrates that supported walking results in different foot trajectories in infants [9] and gait kinematics with more mature levels of coordination [33]. Our skin and ultrasound protocols are not influenced extensively by the infants’ behaviour their inability to follow instructions may mean they move during testing. If this happens the data will be recollected. The skin characterisation devices have not been utilised within this patient group before. However, they have been utilised in published research in adults [21, 27]. Extensive pilot work and comparison to other devices has been undertaken to establish this protocol as feasible, in addition to repeatability work at each laboratory.

It is evident that the infant’s level of emotional and comprehensive development is a challenge to their participation in a standard experimental protocol. Additionally, physical characteristics such as sensitive skin, high subcutaneous fat content and small dimensions should be considered, and standard data collection protocols adjusted. For example, the size and resolution of a pressure platform is designed for testing adults. Infant step length is 25 cm compared to 77 cm in adult walking [34], leading to multiple contacts on the pressure platform. This paired with the variable walking pattern requires manual extraction of collected pressure records, which adds some subjective error to an otherwise automated data extraction protocol. The resolution of the pressure platform is 4 sensors/cm^2^ and the sensitivity measuring range 10–1270 kPa. Therefore, the resolution of infant pressure records are comparatively lower than that achieved from an adult foot with the same technology, however, this resolution is equivalent to adult pressure records from some of the lower resolution pressure platforms utilised for research and therefore can be considered sufficient for comparison. The sensitivity may mean errors in quantification of contact areas (where pressures 10 < kPa are not considered), especially when the soft pliable nature of the infant foot is considered, however this is the current gold standard in this technology. A foot size of approximately 12 cm at age one [5, 14] compared with 24 cm at 17 years of age [35] also allows substantially less surface area for the placement of markers and accurate detection and tracking by 3D motion cameras. The infant’s body composition makes the correct placement of markers on anatomical sites challenging [36]; skin motion artefact may increase due to increased fat mass and less stiff tissue and the non-ossified bones hinder palpation and rigid-body assumptions. Limb dimensions also influence the available distance between markers [37] and body composition the signal achievable through electromyography analysis. Noise and cross-talk from nearby muscles become significant risks and the determination of an appropriate inter-electrode distance is also a challenge. Significant pilot work has enabled the final protocol to minimise such errors and quality checking of data will be continuous.

Despite the challenges addressed above, the importance of this data set warrants the extensive two-site longitudinal study design which is planned. The strengths of this study will be the array of data providing an extensive database of the development of the infant foot as a load bearing structure. The environment and the inclusive approach to the use of data will enable the quantification of the trajectory of variability across these visits in terms of kinematics and pressure. As part of the Great Foundations Initiative, the results of this Small Steps study will contribute to better knowledge and understanding being shared with industry, health care professionals, researchers and parents.

## Abbreviations

EMG: Electromyography
HD: High definition
pSPM: Pedobaragraphic statistical parametric map
